# QRS-T angle in patients with Hypertrophic Cardiomyopathy - A comparison with Cardiac Magnetic Resonance Imaging

**DOI:** 10.7150/ijms.52415

**Published:** 2021-01-01

**Authors:** Christoph Julian Jensen, Moritz Lambers, Behnam Zadeh, Jan Martin Wambach, Kai Nassenstein, Oliver Bruder

**Affiliations:** 1 Department of Cardiology and Angiology, Contilia Heart and Vascular Center, Elisabeth-Krankenhaus Essen, Essen, Germany.; 2 Ruhr University Bochum, Bochum, Germany.; 3 Department of Diagnostic and Interventional Radiology and Neuroradiology, University Hospital Essen, Essen, Germany.

**Keywords:** QRS-T angle, cardiovascular magnetic resonance, late gadolinium enhancement, fibrosis, hypertrophic cardiomyopathy

## Abstract

**Objective:** We sought to investigate the possible association of a wide QRS-T angle on the surface EKG and myocardial fibrosis on contrast-enhanced cardiovascular magnetic (CMR) imaging in patients with hypertrophic cardiomyopathy (HCM).

**Background:** Risk stratification in HCM patients is challenging. Late gadolinium enhancement (LGE) visualizes myocardial fibrosis with unique spatial resolution and is a strong and independent prognosticator in these patients. The QRS-T angle from the surface EKG is a promising prognostic marker in various cardiac pathologies.

**Methods:** 70 patients with HCM obtained a standardized digital 12-lead EKG for the calculation of the QRS-T angle and underwent comprehensive CMR imaging for visualization of fibrosis by LGE. Patients were divided into groups according to the absence or presence of fibrosis on CMR.

**Results:** 43 of 70 patients with HCM showed LGE on CMR following contrast administration. HCM patients with LGE (fibrosis) had wider QRS-T angles as compared to the patient group without LGE (100±54 vs. 46±31; <0.001). A QRS-T angle of 90 degrees or more was a strong predictor (OR 32.84, CI 4.08-264.47; p <0.001) of HCM with LGE.

**Conclusion:** There is a strong association of a wide QRS-T angle and myocardial fibrosis in patients with HCM.

## Introduction

Hypertrophic cardiomyopathy (HCM) is the most common genetic cardiac disease characterized by left ventricular hypertrophy 1. Although the majority of patients are asymptomatic, a small but significant number of patients are at risk of sudden cardiac death (SCD) with a mortality rate of 0.5 to 2% per year 2. HCM is a common cause of SCD in adolescents in general, and competitive athletes in particular 3,4. For these reasons identification of patients at risk and prophylactic defibrillator placement is of utmost importance. Clinical risk factors include massive left ventricular hypertrophy (LVM) of ≥ 30 mm, abnormal blood pressure response to exercise, a family history of HCM with SCD, unexplained syncope and non-sustained ventricular tachycardia on Holter monitoring. Unfortunately the prognostic yield of these risk factors for primary prevention alone or in combination (score) is low 5,6. Late gadolinium enhancement (LGE), however, visualizes myocardial fibrosis with unique spatial resolution and is a strong and independent prognosticator in these patients 7. The QRS-T angle can be easily calculated from every routine digital surface EKG and has shown its prognostic potential in various clinical settings 8,9.

We sought to investigate the association of fibrosis (LGE), the morphologically substrate of life-threatening arrhythmias in HCM, and the QRS-T angle a simple prognostic parameter from routine EKG.

## Methods

We consecutively enrolled patients with known HCM admitted for further risk stratification by CMR to the CV Imaging Section of our Department of Cardiology and Angiology at Elisabeth Hospital Essen Germany. HCM was defined by the presence of left ventricular hypertrophy with an end diastolic septal thickness of 13 mm or more, occurring in the absence of secondary causes such as hypertension, aortic valve stenosis or phenocopy conditions (e.g. storage disease, skeletal myopathy). A standardized 12-lead digital EKG using a Schiller Cardiovit AT 102 plus® was recorded at a speed of 50 mm/s speed with 10mm/mV for the limb and precordial leads. Computerized values of QRS and T-wave axis were automatically given by the Schiller AT 102 plus® software. The frontal QRS-T-angle was calculated as the absolute difference between the frontal QRS- and frontal T-wave axes and expressed as absolute values 10.

CMR images were acquired on a 1.5 Tesla MR System (Magnetom Avanto™, Siemens Medical Solutions, Erlangen, Germany) during end-inspiratory breath-holding using a phased-array receiver coil. Three long axis and 6 to 8 contiguous short axis cine images were acquired by using a steady-state free precession (SSFP: TR 3.2 ms, TE 1.3 ms, matrix 192 × 174, flip angle 80°, bandwidth 930 Hz/pixel, slice thickness 6 mm) sequence covering the entire left ventricle 11. LGE imaging was performed at least 10 minutes following the i.v. administration of 0.15 mmol/kgBW gadoterate meglumine in corresponding long and short axis views by using a segmented inversion-recovery gradient echo sequence (T1w-IR-FLASH: TR 3.1 ms, TE 2.33 ms, matrix 256 × 116, flip angle 30°, bandwidth 300 Hz/pixel, slice thickness 6 mm). The inversion time was adjusted to null the signal of normal myocardium as described previously 12.

LV function was analyzed by outlining epicardial and endocardial borders on the short axis cine sequences. Left ventricular volumes and ejection fraction were derived from contour summation. Maximum wall thickness was evaluated in all end-diastolic short axis cine images. The extent of late gadolinium enhancement was assessed on short axis contrast images by planimetry of hyperenhanced pixels with a signal intensity of 2 standard deviation above normal remote myocardium, calculated by summation of disc method and expressed as percent of total left ventricular mass.

### Statistical analysis

Continuous variables are presented as mean ± standard deviation and compared with the two-way ANOVA test. Categorical variables are presented as numbers and percentages and compared with the chi-square test. Mean QRST angle with 95% confidence interval are presented as a Box-Plot-diagram (**Figure 2**). Differences between groups were analyzed by the two-way ANOVA test. Univariate predictors of the presence of myocardial fibrosis on contrast CMR were analyzed using the chi-square test and expressed as hazard ratio (HR) with 95% confidence interval (CI). Multivariate binary logistic regression was performed to identify independent predictors of myocardial fibrosis on contrast CMR. All variables with an univariate *p*-value <0.05 were included in the final multivariate model. Results of the binary logistic regression model are presented in the same way as the variables in univariate analysis. The correlation between the QRS-T angle and the extent of fibrosis in CMR (fibrosis in percent of LV mass) was tested by pearsons correlation coefficient. All reported *p*-values are two-sided and a *p*-value <0.05 was considered statistically significant. All statistical analyses were performed with IBM SPSS Statistics version 26.0 (IBM Corp., Armonk, N.Y., USA).

## Results

**Figure 1:**
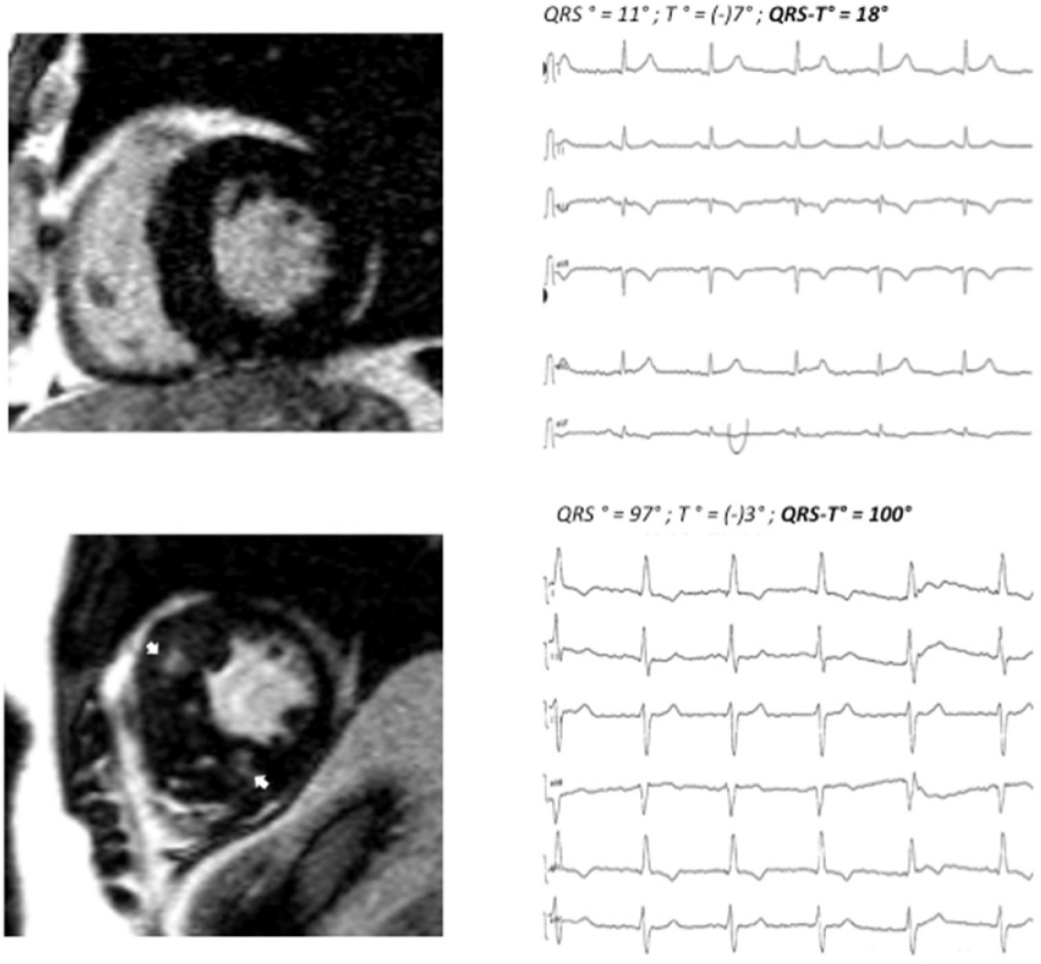
Patient A: basal end-diastolic LGE image showing moderate asymmetric LVH (15 mm MWT), no LGE, QRS-T angle 18°. Patient B: corresponding LGE image showing massive septal LVH (26 mm MWT), typical mid-myocardial LGE pattern in the interventricular septum (arrows). MWT maximum wall thickness, LVH left ventricular hypertrophy. LGE late gadolinium enhancement.

70 consecutive patients (42 male, age 67.7±14.8 years) with HCM were enrolled. Patients were divided into two groups according to the presence (n=43) or absence (n=27) of myocardial fibrosis (LGE) on contrast CMR. Patients with fibrosis had a wider QRS-T angle as compared to patients without fibrosis (100°±54° vs. 46°±31°; <0.001). 24 of 43 patients in the fibrosis group had a QRS-T angle of 90° or more, whereas only 1 of 27 patients in the non-fibrosis group displayed a pathologic QRS-T angle. For clinical examples, also see **Figure 1.**

**Figure 2:**
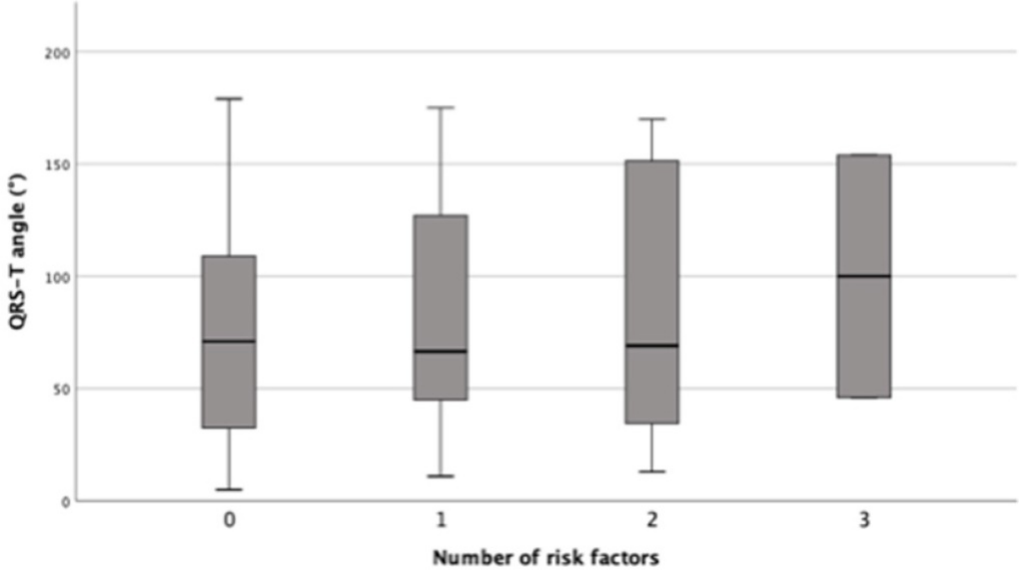
QRS-T angle and number of clinical risk factors (syncope of unknown origin, massive left ventricular hypertrophy ≥30 mm, non-sustained ventricular tachycardia, family history of SCD, LVOT obstruction); differences of mean QRS-T angle between groups were non-significant, p= 0.881.

There were no differences with regards to age, gender, NYHA class and ejection fraction. Patients with myocardial fibrosis had greater maximum wall thickness (21.9±9.1 vs. 17.0±2.8; 0.009). There were no differences in QRS-T angle for each single clinical risk factor (syncope of unknown origin, massive left ventricular hypertrophy ≥30 mm, non-sustained ventricular tachycardia, family history of SCD, LVOT obstruction) between HCM with and without fibrosis (**Table 1**). As the number of risk factors increases, the QRS-T angles increase numerically between groups but missed significance niveau (*p*=0.881) (**Figure 2**).

In a multivariate analysis a QRS-T angle of 90° or more is a strong predictor [HR 32.84 (4.08-264.47); <0.001] of HCM with myocardial fibrosis. There was a moderate correlation between the QRS-T angle on ECG and myocardial fibrosis defined by LGE. With increasing QRS-T angle the extent of myocardial fibrosis increased (R=0.475, *p*<0.001).

## Discussion

Our study shows that in a population of patients with HCM fibrosis visualized by contrast-enhanced CMR is associated with a wide frontal QRS-T angle on the surface EKG.

The QRS-T angle as derived from vectorcardiography represents the spatial angle between the QRS loop (depolarization) and T loop (repolarization) and thus indicates electrical instability. In several cardiovascular patient groups the QRS-T angle has been shown to contribute substantially to risk stratification of SCD 13,14,15,16,17. In addition, the QRS-T angle helps to identify patients with HCM: Potter et al. found that adding the QRS-T angle to standard EKG criteria improved the differentiation of 56 HCM patients from age-matched endurance athletes and healthy controls 18. Cortez et al. used the QRS-T angle to more accurately predict HCM as compared to Italian pre-participation criteria and the Seattle criteria in 130 pediatric patients with HCM 19. Risk stratification by QRS-T angle has been addressed in a study by Cortez et al.: The QRS-T angle performed best for the identification of 20 patients with sustained ventricular arrhythmias out of 100 patients with HCM 20.

Late gadolinium enhancement visualizes HCM with myocardial fibrosis, the border zones of which reflect the morphological substrate for the re-entrant pathways of ventricular tachycardias. Early CMR studies were able to demonstrate a correlation of myocardial fibrosis on contrast CMR and ventricular tachycardia 21,22. More recently the presence and extent of late gadolinium enhanced has been related to outcome in a few studies 23,24. In a group of 1293 HCM patients and a median follow-up of 3.3 years Chan et al. demonstrated a 2-fold increase in SCD for a LGE of ≥15% of LV mass 25.

Shi et al. found that in 66 patients with reduced LV function before ICD placement LGE positive patients had a larger QRS-T angle 26. However and to the best of our knowledge this is the first study about the association of fibrosis CMR and QRS-T angle in HCM.

In our study 24 of 25 (96%) patients with QRS-T angle ≥90° had myocardial fibrosis on CMR, whereas 26 of 45 (58%) patients with QRS-T angle <90° did not display fibrosis. Overall, the extent of fibrosis increased with increasing QRS-T angles on ECG. However, several limitations of our study have to be taken into account. Our study is retrospective analysis in a small patient group, no advanced CMR tissue mapping techniques were used and follow-up was not performed.

In conclusion, a QRS-T angle of 90 degrees or more is strongly associated with advanced disease as indicated by fibrosis on contrast-enhanced CMR. Our study adds to a growing body of literature about the prognostic potential of the QRS-T angle in various cardiomyopathies. Of course prospective studies in a larger HCM patient population are needed to investigate the prognostic impact of the QRS-T angle in this specific group of patients.

## Figures and Tables

**Table 1:** Baseline, ECG and CMR data stratified according presence of myocardial fibrosis

Parameter	Study population (n= 70)	HCM with fibrosis (n= 43)	HCM without fibrosis (n= 27)	*p*-value
Age (y)	67.7±14.8	67.1±14.6	68.5±15.5	0.715
Male gender (%)	60.0	60.5	59.3	1.000
NYHA class	1.4±0.8	1.4±0.8	1.2±0.7	0.283
Syncope of unknown origin (%)	11.4	11.6	11.1	1.000
MWT ≥30 mm (%)	2.9	4.7	0.0	0.519
NSVT (%)	8.6	11.6	3.7	0.394
Family history of SCD (%)	12.9	13.9	11.1	1.000
LVOT obstruction (%)	24.3	20.9	29.6	0.568
**EKG data**				
PQ (ms)	176±41	176±43	177±39	0.876
QRS duration (ms)	98±17	100±17	96±16	0.300
QRS-T angle	79±53	100±54	46±31	<0.001*
**QRS-T angle ≥ 90°**				
**MRI data**				
Ejection fraction (%)	63.2±9.8	62.7±10.4	64.1±8.9	0.558
MWT (mm)	20.0±7.7	21.9±9.1	17.0±2.8	0.009*
Outflow tract obstruction (%)	52.9	46.5	62.9	0.223
Presence of SAM (%)	38.5	34.9	44.4	0.458
Fibrosis % LV Mass	2.6±3.7	4.3±3.8	0.0±0.0	<0.001*

HCM: hypertrophic cardiomyopathy; MWT: maximum wall thickness; NSVT: non-sustained ventricular tachycardia; SCD: sudden cardiac death; LVOT: left ventricular outflow tract; SAM: systolic anterior movement. *Highlights statistical significant differences between groups.

**Table 2:** Predictors of myocardial fibrosis on CMR

Characteristics	HR	95% CI	*p* value
**Univariate analysis**			
Age (years)	0.99	0.96-1.03	0.711
Ejection fraction (%)	0.99	0.94-1.04	0.553
MWT (mm)	1.34	1.11-1.61	0.002
QRS-T angle°	1.03	1.01-1.04	<0.001
**Multivariate analysis**			
MWT (mm)	1.29	1.06-1.04	0.012
QRS-T angle°	1.02	1.00-1.04	0.002
QRS-T angle ≥90	32.84	4.08-264.47	<0.001

MWT: maximum wall thickness.
